# Effects of Mycoplasmas on the Host Cell Signaling Pathways

**DOI:** 10.3390/pathogens9040308

**Published:** 2020-04-22

**Authors:** Sergei N. Borchsenius, Innokentii E. Vishnyakov, Olga A. Chernova, Vladislav M. Chernov, Nikolai A. Barlev

**Affiliations:** 1 Institute of Cytology, Russian Academy of Sciences, 194064 St. Petersburg, Russia; innvish@incras.ru; 2 Kazan Institute of Biochemistry and Biophysics, FRC Kazan Scientific Center, 420111 Kazan, Russia; chernova@kibb.knc.ru (O.A.C.); chernov@kibb.knc.ru (V.M.C.); 3 Moscow Institute of Physics and Technology, 141701 Dolgoprudny, Moscow Region, Russia

**Keywords:** mycoplasma, p53, NF-κB, Nrf2, post-translational modifications, inflammation, hemеoxygenase-1 (HO-1), lipid-associated membrane proteins

## Abstract

Mycoplasmas are the smallest free-living organisms. Reduced sizes of their genomes put constraints on the ability of these bacteria to live autonomously and make them highly dependent on the nutrients produced by host cells. Importantly, at the organism level, mycoplasmal infections may cause pathological changes to the host, including cancer and severe immunological reactions. At the molecular level, mycoplasmas often activate the NF-κB (nuclear factor kappa-light-chain-enhancer of activated B cells) inflammatory response and concomitantly inhibit the p53-mediated response, which normally triggers the cell cycle and apoptosis. Thus, mycoplasmal infections may be considered as cancer-associated factors. At the same time, mycoplasmas through their membrane lipoproteins (LAMPs) along with lipoprotein derivatives (lipopeptide MALP-2, macrophage-activating lipopeptide-2) are able to modulate anti-inflammatory responses via nuclear translocation and activation of Nrf2 (the nuclear factor-E2-related anti-inflammatory transcription factor 2). Thus, interactions between mycoplasmas and host cells are multifaceted and depend on the cellular context. In this review, we summarize the current information on the role of mycoplasmas in affecting the host’s intracellular signaling mediated by the interactions between transcriptional factors p53, Nrf2, and NF-κB. A better understanding of the mechanisms underlying pathologic processes associated with reprogramming eukaryotic cells that arise during the mycoplasma-host cell interaction should facilitate the development of new therapeutic approaches to treat oncogenic and inflammatory processes.

## 1. Introduction

Due to the low complexity and small size of the genome, mycoplasmas are obligatory heterotrophs, and hence, require host cells as a source of nutrients. Through evolution, mycoplasmas have generated many mechanisms to suppress the immune system of host cells to become parasites: see reviews [1,2].

Importantly, mycoplasmas are also contaminants of various cell cultures *in vitro* [3,4]. Among those, the most common (95% of cases) contaminating species are *Mycoplasma arginini*, *M. fermentans*, *M. hominis*, *M. hyorhinis*, *M. orale*, and *Acholeplasma laidlawii* [4,5]. 

In general, Mycoplasma cells localize on the membrane surface of host cells from the outside, but some species of these bacteria are able to penetrate into eukaryotic cells [6] and remain within the host cells [4]. Under certain conditions (mainly in the case of immunodeficiencies), mycoplasmas can damage the host organism, but most often, they behave as “silent parasites” [7]. Nevertheless, seven representatives of the Mollicutes (*M. pneumoniae*, *Ureaplasma urealyticum*, *M. genitalium*, *M. hominis*, *M. fermentans*, *M. penetrans,* and *M. pirum*) are associated with the development of pathology in humans [4]. 

Although mycoplasmas display a high degree of specificity towards host organisms, they are more promiscuous on the level of cell culture contamination *in vitro* [8,9]. Negative effects of mycoplasmas on cell culturing are studied much better compared to their possible impact on host organisms as latent infections. Several studies reported that mycoplasma infections, even at a low level of infection, may contribute to chromosomal instability, chromosomal aberrations and malignancy [10,11,12]. Thus, long-term infection of mouse embryonic fibroblasts with mycoplasmas enhanced “spontaneous” neoplastic transformation elicited by the introduction of the proto-oncogenes H-ras and C-myc [13].

While the titer of mycoplasma infection is low, their presence is asymptomatic for humans and animals [14]. However, if the titer increases, mycoplasmas can directly affect cellular metabolism and physiology of the host organisms by rewiring the process of nutrient consumption. This may result in the generation of reactive oxygen species, which, in turn, triggers genotoxic stress and chronic inflammation. The severity of the effects depends on the ability of mycoplasmas to avoid the host immune control, which allows them to colonize mucosal surfaces and spread to various tissues of the body [1,2,14]. 

Mycoplasmas are available to dampen the consequences of the immune response by blunting the innate immune response and by quickly adapting to stress conditions in the colonized niche. Therefore, to prevent mycoplasma infections, it would be necessary to block the initial stage of infection, thereby preserving the possibility of the full-scale immune response [15,16].

## 2. Mycoplasmas Modulate Inflammatory Response 

The initial stage of mycoplasma infections is related to the attachment of mycoplasmas to membranes of the epithelium of host cells [14]. At the molecular level, the process of the mycoplasma attachment to the surface of mucous cells initially involves the interaction of mycoplasma lipoproteins/lipopeptides or the specific attachment organelles with receptors to the surface of epithelial cells, and in most cases elicits inflammation [14,15,17,18].

The inflammatory reaction is induced by interacting pathogen-associated molecular patterns (PAMPs) with specialized pattern-recognition receptors (PRRs)—Toll-like receptors (TLRs) and NOD-like (nucleotide-binding and oligomerization domain) receptors—expressed in the host cells [16,17]. The process initiates the signaling cascade in the host cell, which determines the specificity of the immune response against the infectious agent [16,19]. 

Many typical bacterial PAMPs (e.g., lipoteichoic acid, flagellin, and some lipopolysaccharides) are absent in mollicutes, and the exact molecular mechanisms of their recognition by the cells of the immune system are not yet well studied. TLRs 1, 2, 4, and 6 were found to bind bacterial LPs [20,21]. It was shown that the macrophage-activating lipopeptide-2 (MALP-2) from *M. fermentans* [22,23,24] binds TLRs; this binding leads to activation of nuclear factor NF-κB [25]. Activated NF-κB induces the expression of pro-inflammatory mediators. It was revealed that the activation of MALP-2 induces the secretion of TNF-α (tumor necrosis factor-α), IL6 (interleukin 6), MIP-1β (macrophage inflammatory protein-1β), GRO-α (growth-regulated oncogene-α), MCP-1 (monocyte chemoattractant protein-1), MIP-1α (macrophage inflammatory protein-1α) [26], CXCL13 (chemokine CXCL13), CXL14 (chemokine CXL14), RANTES (Regulated-on-Activation-Normal-T-cell-Expressed-and-Secreted chemokine) [27], and MIP-2 (macrophage inflammatory protein-2) in monocytes via the activation of the NF-κB-dependent pathway [28]. Similarly, MALP-2 from *M. gallisepticum* R low P47 induces the expression of TNF-α, IL6, and MIP-1β in chicken [27]. Intriguingly, the differential roles of TLR2-2 and TLR6 in *M. gallisepticum*-infected DF-1 cells and chicken embryos were established [29]. Collectively, these data emphasize the evolutionarily conserved nature of the mycoplasmal ligands that elicit the same cellular signaling queues in different mycoplasmas. It is not just the intact cells of mycoplasmas, but also individual lipopeptides from mycoplasmas can induce inflammation [30]. 

Inflammation is a fundamental reaction of the innate immune system, leading to a protective response from the organism to the stimuli, including those stimuli associated with infectious agents [31]. However, in the case of long-acting stimuli and persistent infection, the release of pro-inflammatory factors, reactive oxygen species (ROS), nitric oxide (NO), etc., can lead to “hyper-inflammation” with severe tissue damage as a consequence [32]. This creates the need to study the complex controlling networks of the relevant processes. It has now been established that the inflammatory response is modulated by a number of transcription factors and cellular pathways [33]. The classic NF-κB pathway promotes the production of ROS and pro-inflammatory mediators [34], whereas the NF-E2-related factor 2 (Nrf2) is a key transcription factor that reacts to oxidative stress conditions and protects cells from ROS and inflammation by inducing anti-inflammatory, cytoprotective factors, including HO-1 [35].

The gene for HO-1 is known to contain a multitude of stress-activated response elements, including antioxidants (AREs) in the 5’ untranslated region [36]. A key factor in the AREs-mediated induction of antioxidant proteins and the regulation of inflammatory responses to a variety of stimuli is Nrf2 [37,38]. NF-κB-mediated transcription reduces Nrf2 activation by downregulating ARE-dependent gene transcription, and decreases free CREB binding protein (CBP) by competing with Nrf2 for CBP [39]. NF-κB also enhances the recruitment of histone deacetylase (HDAC) to the ARE region, and therefore, interferes with the transcriptional facilitation of Nrf2 [40]. The activation of Nrf2 suppresses the pro-inflammatory cytokine-induced expression of adhesins in endothelial cells [41]. In Nrf2-deficient mice, compared to wild-type mice, the endotoxin treatment causes a higher level of expression of pro-inflammatory cytokines [42]. Finally, Rushworth et al. [43] have demonstrated that, in response to lipopolysaccharide (LPS), macrophages synthesize cytoprotective proteins, including HO-1 and NAD(P)H quinone dehydrogenase 1 (NQO1), which protect the host cells from an excessive inflammatory response induced by the activation of the NF-κB pathway. Nrf2 regulates the LPS-induced production of HO1 and NQO1, thereby modulating the pro-inflammatory response induced by LPS. It has been shown that lipoproteins and lipopeptides of some mycoplasmas can also exhibit similar properties [44,45,46,47]. Such information was obtained with respect to the membrane lipoproteins of *M. pneumoniae*, *M. gallisepticum* and *M. genitalium* – LAMPs (lipid-associated membrane proteins), as well as the lipopeptides of *M. fermentans*—MALP-2 (macrophage-activating lipopeptide, the precursor of which is the lipoprotein MALP-404). MALP-2 is the first lipopeptide in which binding to Toll-like receptors (TLR2/6) has been established. It has also been shown that the lipopeptide conformation has a significant effect on the lipopeptide’s interaction with the receptor, and on the level of the NF-κB stimulation [23,48]. 

Ma et al. have shown that MALP-2, like LPS, induces the expression of HO-1 and increases the enzyme activity in vitro - in THP-1 cells [44]. It has been found in the study that the MALP-2-dependent expression of HO-1 is mediated by mitogen-activated protein kinases (MAPKs) and Nrf2, and modulates the cyclooxygenase-2 (COX-2) expression induced by MALP-2. MALP-2 activates the translocation of Nrf2 into the nucleus, which further induces HO-1 expression, whereas suppressing Nrf2 via siRNA attenuates the expression of the protein. The expression of HO and the enzymatic activity of this protein in the experiments of the authors increased in response to MALP-2. The increase was shown to be directly dependent on the MALP-2 concentration. At the same time, it was revealed that HO-1 suppresses the MALP-2-induced COX-2 expression in THP-1 cells. 

It is believed that the expression of HO-1 in monocytes mediates the anti-inflammatory effect by modulating their functions in the inflammatory response [49]. Ma et al. [44] showed that inhibitors (SB203580, PD98059, SP600125) of the MAPKs (mitogen-activated protein kinases) signaling pathway proteins, the central pathway that regulates a wide spectrum of cell response to various stimuli, including stress, can block the HO-1 expression induced by MALP-2. The mechanisms of HO-1 induction by various stimuli are widely discussed [50,51,52,53]. However, Ma et al. [44] added a new aspect to this discussion when they showed that, in the case of the mycoplasmal lipopeptide, MALP-2, HO-1 expression may depend on the activation of MAPKs and Nrf2, and according to the existing notions on signaling pathways, HO-1 can further regulate the response of monocytes to MALP-2 by suppressing an excessive inflammatory response to the corresponding stimulus. The ability of this lipopeptide to induce Nrf2 translocation into the nucleus, as well as the expression of HO-1, and inhibit the expression of COX-2 and, consequently, prevent an excessive inflammatory response, seems very valuable in terms of its pharmacological utility. The derivatives of MALP-2 and its analogues, which do not cause inflammatory responses to host cells, may prove promising for the treatment of diseases associated with chronic inflammation.

**Figure 1:**
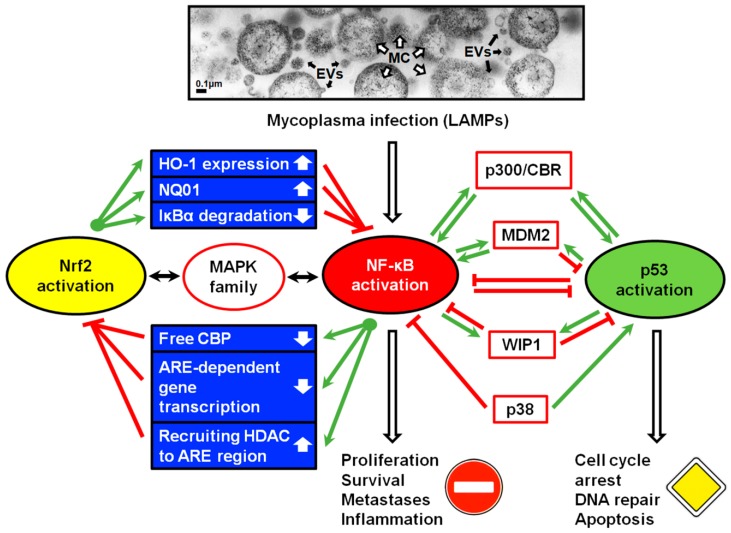
A road map of the cellular processes influenced by mycoplasma infection. When cells are infected with mycoplasmas, a number of signaling pathways involved in the regulation of tumor transformation and progression are affected. A particular role in the process may belong to mycoplasmal lipid-associated membrane proteins (**LAMPs**) that are present in mycoplasma cell (**MC**) membranes and extracellular vesicles (**EVs**). TEM of *Acholeplasma laidlawii* PG8a ultrathin sections (as an example of mycoplasmal infection) is on the upper part of the scheme, proposed “cross-talk” between **Nrf2** and **NF-kB** is on the left side of the scheme, and between **NF-kB** and **p53,** on the right side. Green arrows are induced events; red lines are the resulting inhibitory events; black arrows represent the concerted modulation. **ARE** = antioxidant response elements; **CBP** = CREB binding protein (CREB = cAMP-response element-binding protein); **HDAC** = histone deacetylase; **HO-1** = heme oxygenase-1; **IkBα** = nuclear factor of kappa light polypeptide gene enhancer in B cells inhibitor, alpha; **MAPK** = mitogen-activated protein kinases; **MDM2** = mouse double minute 2 homolog, also known as E3 ubiquitin-protein ligase; **NF-κB** = nuclear factor kappa-light-chain-enhancer of activated B cells; **NQO1** = NAD(P)H quinone dehydrogenase 1; **Nrf2** = NF-E2-related factor 2; **p38** = a class of mitogen-activated protein kinases (MAPKs) that are responsive to stress stimuli; **p300/CBP** = associated factor (PCAF), also known as acetyltransferase 2B (KAT2B); **WIP1** = wild-type p53-induced phosphatase 1.

The ability to modulate inflammatory responses through the activation of Nrf2 has also been demonstrated in relation to the surface lipoproteins (LAMPs) of *M. pneumoniae* [47] and *M. genitalium* [46]. The data obtained by Hu et al. [47] indicate that the LAMPs of *M. pneumoniae* activate NF-κB signaling pathways, which determine pro-inflammatory effects, but that mycoplasma factors also activate the Nrf2, which determines the anti-inflammatory effects. It is possible that there is ‘cross-talk’ between Nrf2/ARE and NF-*κ*B signaling pathways in response to LAMPs. All regulators of the driving forces behind the host cell outcome remain to be studied (see Figure 1). 

Recently, it became known, that the protective effects of the flavonoid baicalin were associated with the up-regulation of the Nrf2/HO-1 pathway and suppression of the NF-κB pathway in the spleen of chickens infected with *M. gallisepticum* [54]. The baicalin treatment also efficiently inhibited oxidative stress and apoptosis via activation of the Nrf2 signaling pathway, and could protect the chicken thymus from infection-mediated injury [55].

## 3. Mycoplasmal Infections Promote Tumor Transformation

Whether mycoplasma infections can provoke chromosomal aberrations in host cells is still debated [56]. In line with this is the discovery that certain mycoplasma strains have the ability to induce karyotypic changes in chronically infected cells [57,58]. Eventually, these chromosomal aberrations may induce cellular neoplastic transformation. 

Several studies have analyzed transcriptomes of mycoplasma-infected cell cultures. They showed that there were dramatic changes in the gene expression profiles of host cells, caused by the mycoplasmas. Gene ontology analysis has revealed that the genes affected by mycoplasmas included oncogenes, tumor suppressor genes, cytokines, receptors, and components of various signaling pathways [59,60,61,62]. 

A few hours post-infection, changes in gene expression were already noticeable, with the long-term cultivation of infected cells promoting their irreversible transformation. In accordance with this, following the prolonged co-cultivation (19 weeks) of benign epithelial human prostate cells (BPH-1) with *M. genitalium* or *M. hyorhinis* the neoplastic transformation of the BPH-1 was observed [63].

Malignant transformation of these cells was validated by xenograft experiments in nude mice. Increases in the numbers of chromosomal aberrations and polysomy were revealed by karyotypic studies. These data indicate that mycoplasmas probably act as contributing factors to the development of both human and animal cancers. More evidence of the tumorigenic role of mycoplasmas stems from the fact that patients suffering from prostate cancer are infected three times more often with *M. hominis* and *U. urealyticum* than patients with benign prostatic hyperplasia [64,65]. However, without conducting any large-scale epidemiologic correlation studies it would be impossible to prove whether mycoplasma infection and a predisposition to cancer are connected. 

Signaling arbitrated by the binding of ligands with TLRs typically results in the activation of NF-κB and in the commencement of the cell’s anti-apoptotic program (Figure 1). Alongside TLR signaling, there are numerous other pathways that are involved in cancer, which come together for the initiation of NF-κB [66,67]. 

In this regard, it is essential to note that multiple published reports submit that mycoplasmas are able to avert apoptosis or on the contrary, induce cell death [10,68,69]. Five species of *Mycoplasma* were subject to a more detailed examination, and only *M. fermentans* and *M. penetrans* were able to effectively support the nonstop growth of 32D cells (a mouse myeloblast-like cell line derived from the bone marrow) after the elimination of IL-3.

The infection of cells with *M. fermentans* caused a stronger effect than *M. penetrans*. Contrastingly, *M. hominis* and *M. salivarium* served to accelerate the apoptosis of 32D cells; while *M. genitalium* had no noteworthy effect on cell death. It was concluded that varying species of *Mycoplasma* differentially affect the release of key pro-apoptotic genes and hence result in either the promotion or repression of apoptosis [70]. In response to acute inflammation, cells elicit the strong, short-term, reversible activation of NF-κB. Such a severe retort leads to the successful elimination of the infectious agent (or another stimulus), subsequently followed by the termination of inflammation and by the restoration of damaged tissue.

Thereafter, the instigation of NF-κB is ended via a feedback mechanism that involves the induction of IκB, an inhibitor of NF-κB. Contrastingly to the acute response, the mechanism of NF-κB activation as a consequence of chronic inflammation is moderate but sustained. The latter may be associated with a decreased level of the inhibitor IkBa’s release, or with the reactivation of NF-κB caused as a consequence of the constitutive presence of the PAMP (pathogen-associated molecular pattern) or DAMP (danger-associated molecular pattern), which results in a prolonged oscillation of the activity of NF-κB. As a matter of fact, it is this prolonged activation of NF-κB that serves to cause a state of chronic inflammation [71,72,73]. In accordance with this, it was shown that NF-κB is constitutively activated in most tumors [74] and generally functions as an oncogene with a distinct anti-apoptotic effect [75].

## 4. NF-κB and p53 Pathways in Eukaryotic Cells

A well-known adversary to NF-κB is p53, which, debatably, is the major tumor suppressor in humans [76]. Numerous studies have established a solid connection between chronic inflammation and tumor progression. The consequence of the mutual regulation between anti-apoptotic NF-κB and pro-apoptotic p53 serves a major role in determining the fate of the cell (review: [77]), (see Figure 1). Genetic or pharmacological inhibition of constitutively active NF-κB in different tumor cell lines leads to the activation of the p53 function, as well as to tumor cell death via p53-dependent apoptosis [75]. Due to the fact that inflammation can suppress p53 activity, it is plausible that the molecular basis for the tumorigenic effect of chronic inflammation is mediated by activated NF-κB, which gains the ability to suppress genome protective functions of p53 [77]. 

The most crucial role of the transcription factor p53 is to reduce oncogenic activity [78]. The suppression of p53 activity is imperative for the transformation of mammalian cells by driver oncogenes. For instance, in rodent cells, the only thing preventing the induction of fully transformed phenotypes in fibroblasts with activated RAS activity is p53 [79]. Notably, abolishing the function of p53 is a crucial criterion for the successful replication of certain viruses, such as human papillomavirus (HPV) and adenoviruses. HPV infection causes the physical degradation of p53 by binding to the viral protein E6 [80]. If p53 is insufficiently inactivated, the virus is prevented from replicating, as a result of the rapid apoptotic response in the cells of the host [81]. 

The similarity between viral and mycoplasma infections is highlighted by the fact that rodent fibroblasts undergo an oncogenic transformation with H-RAS in the presence of *M. arginini*, whereas mycoplasma-free cells undergo a permanent, p53-dependent cell cycle arrest [82]. 

In the cited work, the authors state that in their experiments, mycoplasma infection was as effective as the shRNA-mediated knockdown of p53 expression in fibroblasts of rodents, which makes these cells susceptible for RAS-induced transformation. This indicates that the mycoplasma functionally acts as an oncogene, i.e., suppressing p53 and cooperating with RAS during cell transformation. The noted carcinogenic and mutagenic effects of mycoplasmas can be linked to the inhibition of p53 functions due to the activation of NF-κB [78,82]. 

The NF-κB signaling network is the primary controller of the immune response to outside stresses, including infection with viruses, parasites, mycoplasmas, etc. [73]. Not surprisingly, the NF-κB pathway is frequently aberrantly regulated in cancer. This is similar to another critical stress-dependent pathway, the p53 signal transduction pathway, but the outcome of this dis-regulation is different. In contrast to p53, which becomes inactivated upon cellular transformation, NF-κB is activated by persisting infections and usually takes the role of an oncogene with a strong anti-apoptotic effect [74]. 

As mentioned earlier, constitutively active NF-κB is one of the main causes of the functional inactivation of p53 in tumors [75]. Thus, the most commonly observed interaction between NF-κB and p53 is antagonistic [83,84]. Several mechanisms of the NF-κB-dependent inactivation of p53 have been reported to date. Amongst the known models that explain the NF-κB-mediated negative regulation of p53, one model is executed via the up-regulation of Mdm2, a major inhibitor of p53, which is an E3 ubiquitin ligase directing degradation of p53 [85,86]. Notably, a protracted activation of NF-κB creates a state of chronic inflammation and thus plays a crucial role in predisposition to cancer [73,85]. 

*M. arginini* was shown experimentally to concomitantly suppress the function of p53 and constitutively activate NF-κB in cultured cells. This double effect imitates the common feature of tumor cells during their malignization, which causes uncontrolled growth, genomic instability, and resistance to apoptotic stimuli [77,82]. 

How does mycoplasma activate NF-κB? One explanation can be that mycoplasmas via the R-Pam2 lipopeptide membrane components stimulate TLR2/TLR6 receptors [21]. Their binding activates signaling pathways that stimulate NF-κB release. In sequence, NF-κB then brings about the release of pro-inflammatory cytokines and chemokines. However, the exact molecular mechanisms responsible for the stimulation of NF-κB by mycoplasmas, and the subsequent inhibition of p53-dependent functions remain to be elucidated.

## 5. Molecular Mechanisms of p53 and NF-κB Antagonism

### 5.1. The Contest for p300 Acetyltransferase

p53′s or RelA’s ability to trigger transcription, primarily relies on the relative level of the histone acetyltransferase protein (p300/CBP), which is an essential co-activator for both transcription factors [87,88]. It is imperative to note that the experimental findings of multiple laboratories, including ours, demonstrated that this effect was observed with endogenous levels of these transcription factors, signifying its biological significance and importance.

Furthermore, RelA can repress p53 by hijacking the major transcriptional co-activator of p53, histone acetyltransferase p300/CBP [89]. RelA binds two specific regions in the p300 co-activator, located in the N- and the C-termini of the protein. Notably, the same regions of p300 also interact with p53. In response to inflammatory signals, the NF-κB complex translocates to the nucleus and activates the transcription of its target genes using p300/CBP. However, upon stress, the level of p53 in the nucleus is increased resulting in the accumulation of p300/CBP at the regulatory regions of p53-dependent genes, and hence, the down-regulation of the NF-κB response. Importantly, forced overexpression of p300 was shown to alleviate the p53-mediated repression of RelA, emphasizing the fact that p300/CBP is likely a restricting influence for both transcription factors.

Importantly, the acetylation of both p53 and RelA proteins is modulated by another post-translational modification, lysine methylation, which modulates yet another covalent modification, ubiquitinylation that in turn competes with acetylation for the target lysines. Lysine-specific methyltransferase (KMT) Set7/9 is the major responsible enzyme mediating this modification. Functionally, lysine methylation of RelA on K314 and K315 and p53 on K372 produces opposite effects, i.e., RelA undergoes degradation, and on the contrary, p53 is stabilized by Set7/9-dependent methylation [76,90,91,92]. It will be important to see whether its activity or the protein level is modulated by mycoplasma infections since Set7/9 has been shown to undergo Mdm2-dependent ubiquitination.

### 5.2. p53- and NF-κB- Targeting Micro-RNAs

It is established that both p53 and NF-κB exert their roles as master-regulators of gene expression via both the control of protein-coding genes and the control of non-coding RNAs [93,94]. In general, micro-RNAs are between 20–25 nt in length, and are part of the small double-stranded RNA group [95]. Consequently, it is probable that these two transcription factors can weaken each other by altering the expression of specific micro-RNAs. Supporting this hypothesis are several studies that demonstrate an inverse correlation between the levels of NF-κB-dependent micro-RNAs and p53. Specifically, p53 was targeted by miR-146a, whose expression was under the control of NF-κB [96].

Furthermore, the release of miR-125, which also focuses on p53, was increased upon the triggering of NF-κB by UV irradiation [97]. MiR-155 is another oncogenic miRNA that targets p53 in an NF-κB dependent manner and is prevalent in blood malignancies [98]. It will be fascinating to see whether miR-150, another p53-targeting miR, is also controlled by NF-κB. Functionally, the reduction of miR-150 caused stabilization of p53 and a reduction of NF-κB [96]. The opposite effect is also true; specifically, p53 instigation causes elevated levels of miR-192, miR-194, and miR-215, whose release may target (directly or indirectly) the activity of NF-κB, and thus attenuate the inflammatory response [99]. Taken together, it is clear that cross-talk between p53 and NF-κB can be actualized via micro-RNAs. Thus, it is evident that this research ought to be investigated further.

## 6. Effects of Mycoplasmas on Expression of Micro-RNAs

The data described above raise an intriguing possibility - that mycoplasmas are influencing host cells via modulating their micro-RNA expression. This may be a new method used by parasites to reprogram host cells, but unfortunately, there are only a few publications on this topic available to date.

For example, micro-RNAs linked with a serum antibody response to *M. bovis* in beef cattle were discovered. There were 21 micro-RNAs meaningfully connected to the infection, including bta-let-7b, bta-miR- 24-3p, bta-miR- 92a, and bta-miR-423-5p [100]. One of the most pathogenic mycoplasmas, *M. gallisepticum*, was reported to trigger the release of gga-miR-101-3p in the lungs of chicken embryos infected with MG. Furthermore, one of the targets for gga-miR-101-3p is the gene known as the enhancer of zeste homolog 2 (EZH2). This micro-RNA binds to the 3’ untranslated region (3’-UTR) of the EZH2 gene and attenuates its expression [101]. Another chicken micro-RNA, gga-miR-99a, which targets SMARCA5, was detected as enhanced in response to *M. gallisepticum*. Functionally, the over-expression of gga-miR-99a caused the decrease of cell proliferation in chicken [102]. Importantly, the miR-99 family of microRNAs correlated with radiation sensitivity in human cells. These microRNAs focused on the SWI/SNF chromatin remodeling factor SNF2H/SMARCA5, a component of the ACF1 complex. Importantly, miR-99a reduced BRCA1 localization to sites of DNA damage, thus decreasing the rate and overall efficiency of homologous recombination and non-homologous end-joining [103]. Micro-RNA gga-miR-16-5p, when upregulated, could decrease multiplication, cycle progression, and increase apoptosis of *M. gallisepticum* infected chicken DF-1 cells, at least partly through directly targeting PIK3R1 and inhibiting PI3K/Akt/NF-κB pathway to exert an anti-inflammatory effect [104]. As a last example, upon *M. gallisepticum* infection, up-regulation of miR-130b-3p activates the PI3K/AKT/NF-κB pathway, and facilitates cell proliferation and the cell cycle via downregulating PTEN (phosphatase and tensin homolog) [105]. Evidently, more work is required in order to thoroughly uncover the effects of mycoplasmas on the expression profiles of micro-RNAs in host cells of differing origins. The role of extracellular vesicles derived from host cells and mycoplasmas that contain sRNAs and mediate the interaction between pro- and eukaryotes [106,107], remains to be studied.

## 7. Conclusions

On the molecular level, pathogenic infection of host cells with mycoplasma elicits stress, production of ROS culminating in activation of typical stress-response pathways, including the one regulated by p53. However, mycoplasmas employ various mechanisms to disguise themselves from the surveillance by inhibiting p53. Since p53 is considered as a guardian of the genome, it is not surprising that mycoplasma can contribute to tumorigenesis [108]. Moreover, since p53 is regulated mostly by post-translational modifications, mycoplasmas hijack relevant enzymes (e.g., histone acetyltransferase p300/CBP) to blunt the p53-dependent response. In this respect, it would be interesting to see whether inhibitors of HDAC1/2 that deacetylate p53 can restore the activity of p53.

Similarly, inhibitors of enzymes that trigger p53 degradation, including specific E3 ubiquitin ligases (MDM2, Pirh2, etc.) and MAP kinases should also be tested as potential anti-mycoplasmal agents.

Besides, mycoplasmas are capable of modulating inflammatory responses through the activation of a transcription factor Nrf2. The mycoplasma lipopeptide/lipoprotein-induced expression of HO-1 tends to suppress inflammation. Mycoplasma membrane lipoproteins (LAMPs), along with lipoprotein derivatives (lipopeptide MALP-2) can shift “cross-talk” between the pro- and anti-inflammatory signaling pathways in favor of anti-inflammation. Therefore, the LAMPs and MALP-2 derivatives should be examined for their therapeutic potential to treat chronic inflammation-associated diseases. The study of the NF-κB/Nrf2/p53 signaling network is important for elucidating mechanisms underlying pathologic processes associated with reprogramming eukaryotic cells that arise during the mycoplasma-host cell interaction. This can facilitate the development of new approaches for the treatment of inflammatory processes relevant to both human and livestock therapy.
